# Proton therapy for pancreatic cancer: real-world single-center experience of efficacy, toxicity, and predictors of outcome

**DOI:** 10.3389/fonc.2026.1710943

**Published:** 2026-02-26

**Authors:** Philipp Lishewski, Zelal Geyik, Linda Agolli, Kerem Tuna Tas, Houmam Anees, Klemens Zink, Fabian Eberle, Anja Rinke, Hilke Vorwerk, Thomas Held, Daniel Habermehl, Sebastian Adeberg, Ahmed Gawish

**Affiliations:** 1 Department of Radiotherapy and Radiation Oncology, Marburg University Hospital, Marburg, Germany; 2 Department of Radiotherapy and Radiation Oncology, Philipps University Marburg, Marburg, Germany; 3 Marburg Ion-Beam Therapy Center (MIT), Department of Radiotherapy and Radiation Oncology, Marburg University Hospital, Marburg, Germany; 4 Department of Radiotherapy and Radiation Oncology, University Cancer Center (UCT) Frankfurt – Marburg, Marburg, Germany; 5 Department of Radiation Oncology, Justus-Liebig-University Giessen, University Hospital, Giessen, Germany; 6 Department of Trauma, Hand and Reconstructive Surgery, University Hospital Giessen and Marburg, Giessen, Germany; 7 State offensive for the development of scientific and economic excellence (LOEWE) Research Cluster for Advanced Medical Physics in Imaging and Therapy, (ADMIT), University of Applied Science (TH) Mittel Hessen University of Applied Sciences, Giessen, Germany; 8 Institute of Medical Physics and Radiation Protection, University of Applied Sciences, Giessen, Germany; 9 Department of Gastroenterology and Endocrinology, Philipps University Marburg, Marburg, Germany; 10 Department of Radiation Oncology, Heidelberg University Hospital, Heidelberg, Germany; 11 National Center for Tumor Diseases (NCT), NCT Heidelberg, a Partnership between German Cancer Research Center (DKFZ) and University Medical Center Heidelberg, Heidelberg, Germany

**Keywords:** chemoradiation, chemotherapy, image guided radiation therapy (IGRT), pancreas cancer, proton therapy

## Abstract

**Background:**

Pancreatic cancer has a poor prognosis, with five-year survival rates of 10-13%. Surgery is the only curative option, though not feasible for many patients. Radiation therapy is crucial but limited by surrounding radiosensitive organs. Proton therapy provides improved dose distribution and higher biological effectiveness; however, clinical data comparing it to conventional photon therapy remains scarce.

**Methods:**

We retrospectively analyzed 41 patients with unresectable or recurrent pancreatic cancer treated with curative-intent proton therapy at Marburg Ion Beam Treatment Center. Median patient age was 69.5 years; 58.5% were male, 70.8% had T3/4 tumors, and 73.2% presented at stage III. Median delivered dose was 54.0 GyE (median BED: 64.0 GyE) over 30 fractions. Most (95.1%) received proton therapy alone, 68.3% with concurrent chemotherapy. Outcomes evaluated included overall survival (OS), local recurrence-free survival (LRFS), progression-free survival (PFS), and CTCAE v5.0-assessed toxicities.

**Results:**

Proton therapy showed good tolerability with minimal toxicity (early grade ≥III: 2.4%; late grade III: 4.9%, no late grade IV/V). Local recurrence occurred in 13 patients (all within 6 months), and overall progression was noted in 33 patients (mostly within 1 year). Salvage RT correlated with worse PFS on univariate analysis (p=0.03), though non-significant in Cox regression (p=0.06). Adjuvant chemotherapy showed a non-significant trend toward improved OS (HR = 0.53, p=0.08). No dose-related factors influenced local recurrence.

**Conclusions:**

Proton therapy for pancreatic cancer was well tolerated, though outcomes remained modest compared to recent studies utilizing higher doses and optimized protocols. Low toxicity supports proton therapy’s theoretical advantages. Dose escalation and protocol optimization may enhance outcomes. Prospective studies with larger cohorts are warranted.

## Introduction

To this day, pancreatic cancer (PC) remains one of the most devastating cancer types. While only affecting around 14 per 100, 000 persons per year (1), the five year survival rate is estimated to be around 10% - 13% (1, 2). While Surgery remains the only potentially curative treatment option, mostly combined with neoadjuvant or adjuvant chemotherapy (Cx) (3–6), the role of Radiotherapy (RT) or radiochemotherapy (RCx) is debated.

Adjuvantly, the only evidence suggesting a benefit from RCx stems the 1980s (7). Two later trials denied advantages of adjuvant RCx over Cx (8, 9). In the neoadjuvant setting, the PREOPANC trials established both Cx and RCx, not only for borderline resectable PC (BRPC), as viable treatment options (10, 11) with the first PREOPANC-trial showing increased overall survival (OS) rates and R0-resection rates compared to surgery alone (10, 11). Most studies employed lower RT doses or split course regimens. However, it is assumed that PC benefits from dose escalation (12). Besides SBRT, proton therapy (PT) represents a way of dose escalation through its higher linear energy transfer (LET) (13, 14) while reducing dose at organs at risk (OAR) thanks to the Bragg-beak phenomenon (15). Furthermore, the relative biological effectiveness (RBE) of proton therapy (PrT) is estimated to be higher with a value of at least 1.1 (16) and possibly exceeding 2.0 in the distal portion of the Bragg peak (15).

To this date, clinical data on the use of PT remains scarce, though existing evidence is promising. A Japanese study showed a 1-year OS of 76.8% using PT and concurrent Cx for unresectable or BRPC (17). Comparable results were achieved for C12-radiotherapy in case of recurrent disease after resection in Terashima et al.’s work (17). Although this data is promising, head-to-head comparisons between photon and particle therapy and between those regimens and their interplay with chemotherapy are missing and further clinical studies are underway (18). Due to this, efforts have been made to standardize the biological effective dose (BED) of chemoradiation by taking sensitization and cytotoxic Cx-effects as well as different types of radiotherapy and fractionation into account (19). These approaches can aid to better understand and compare therapeutic regimens for PC as data remains scarce and could be of special interest given the impact that chemotherapy seems to have on the course of PC.

In a prior analysis from our institution, feasibility and dosimetric aspects of PrT were reported in a cohort of 25 patients [20]. The present study builds upon that foundation by analyzing an expanded cohort, with a focus on clinical outcomes, toxicity, and the introduction of biologically effective chemotherapy dose (BECD) modeling to assess dose–response relationships.

## Materials and methods

After obtaining a positive ethic vote (NR 24-234-RS) we screened all patients who received PrT for PC at the Marburg Ion Beam Treatment Center (MIT). We excluded patients who received photon therapy only or therapy with C12-therapy, under-aged patients (>18 years), cases with re-irradiations, non-adenocarcinoma-histology or secondary neoplasms as well as patients with sub-ablative doses treated in palliative intention. We included patients with unresectable or recurrent PC referred to salvage RT treated in curative intention with PrT. Patients who received a proton boost as part as of a combined photon-proton therapy were also included. Thus, out of 53 patients screened, 41 patients were included.

Prior to radiotherapy, eligible patients received counselling from a radiation oncologist. 4D-CT consisting of individual breathing phases was utilized for treatment planning, with a slice thickness of 3 mm. Immobilization was aided by customized vacuum mats. Single beam-optimized or intensity-modulated proton beam plans were calculated (Syngo.via RT Planning, Siemens Healthineers, Erlangen, Germany), with a minimum of two 45-degree proton beams used for PrT-delivery via raster scanning system (20). Treatment planning was based on CT and MRI imaging. The PTV-dose was prescribed to the 95% isodose, while a maximum dose of 107% was accepted. Based on the 4D-CT an internal target volume (ITV) was delineated by expansion of the gross tumor volumes (IGTV) from all breathing phases. Lastly, the planning target volume (PTV) was generated with individual margins depending on each patient’s anatomy and immobilization. Image guided radiotherapy (IGRT) was performed daily using X-ray imaging. For patients with lymphonodal involvement, involved lymph nodes were included into the target volumes. Routinely, the peripancreatic neural plexuses were also included in the target volume. The first follow-up examination occurred six weeks after RT, the first MRI or CT-scan (depending on modality used pre-operatively) took place after three months and was repeated every three to six months thereafter. Our institutional RT-doses ranged from 45 – 63GyE. Typically, higher doses were applied for primary RT and/or in cases enough distance to neighboring OARs allowed dose-escalation with X-ray IGRT. Conversely, the dose was reduced in case of adjacent OARs, suboptimal interfractional positioning or for excessively large PTVs.

To account for differences in fractionation (n = number of fractions) and dose per fraction (d), biologically effective dose (BED) was calculated according to the linear-quadratic model as:

$BED=n\cdot d\cdot RBE\cdot \left(1+\frac{d\cdot RBE}{\text{α}/\text{β}}\right)$with α/β=9.5 for PC (21). A RBE of 1.0 was assumed for photon therapy, 1.1 for proton therapy (16). For combined proton and photon treatments, the total BED was computed by addition of the BED contributions of each modality according to the respective fractions applied with each modality. Subsequently, we added variables for sensitizing chemotherapy cycles during the RT and for independent cell kill through those Cx -cycles to achieve an estimation of the biologically effective chemotherapy dose (BECD) (19, 21, 22)


$$BECD=\left(n-m\right)\cdot d\cdot RBE\cdot \left(1+\frac{d\cdot RBE}{\text{α}/\text{β}}\right)+m\cdot d\cdot RBE\cdot s\cdot \left(1+\frac{d\cdot RBE\cdot s}{\text{α}/\text{β}}\right)+\frac{1}{\text{α}}\cdot \ln\left(\frac{\ln\left(TCP_{rad}\right)}{\ln\left(TCP_{rad+chemo}\right)}\right)$$

Here, we assumed a sensibilizing factor s = 1.316 for Gemcitabine, which was the only Cx administered during RT in this cohort with a standard dose of 300 mg/m^2^ weekly. Additionally, the number of concomitant Cx-cycles (m) were included in the model. Differing from the first formula, here the second term accounts for the increased biological effect of radiation during fractions given with concurrent chemotherapy, modeled via a sensitization factor s. α was assumed to have a value of 0.015Gy^−1^ (21). The third term estimates the additional cell kill from chemotherapy alone, based on the difference in tumor control probability (TCP) between radiotherapy alone and chemo radiotherapy (19, 22). To quantify the independent cytotoxic effect of chemotherapy, we assumed a spread of possible increases in tumor control probability (TCP) from ~0.50 with radiotherapy alone to ~0.60 or ~0.70 with gemcitabine-based chemoradiotherapy, extrapolated from outcomes reported in prospective LAPC trials showing ranging results (23–25).

For statistical Analysis SPSS version 29 from IBM (26) and GraphPad Prism version 10 (27) were used with a significance level set to p<.05. For metric variables, the Shapiro-Wilk test was used to check for normal distribution. In cases of normal distribution, t-tests for independent samples were performed to check for imbalances between patients receiving primary vs. adjuvant RT. Otherwise, the Mann-Whitney U-test was employed. For categorical variables, this was tested using Chi-square tests or Fisher’s exact test in cases with< 5 counts per cross table cell. The Kaplan-Meier method was employed to depict overall survival (OS), local recurrence-free survival (LRFS), and progression-free survival (PFS) after radiotherapy (RT). PFS was defined as the time from RT to any locoregional recurrence, distant metastasis, second primary malignancy, or death to any cause. LRFS referred to the time of tumor recurrence within the planning target volume (PTV) previously irradiated. Individual log-rank and Cox testing was performed to identify predictors of these clinical endpoints. Log-rank tests were used for categorical variables, while Cox regressions were computed for all variables allowing an estimation of hazard ratios under the proportional hazards assumption. Multivariate Cox proportional hazards models were employed to confirm predictors of PFS and OS.

## Results

### Patient characteristics

**Figure 1:**
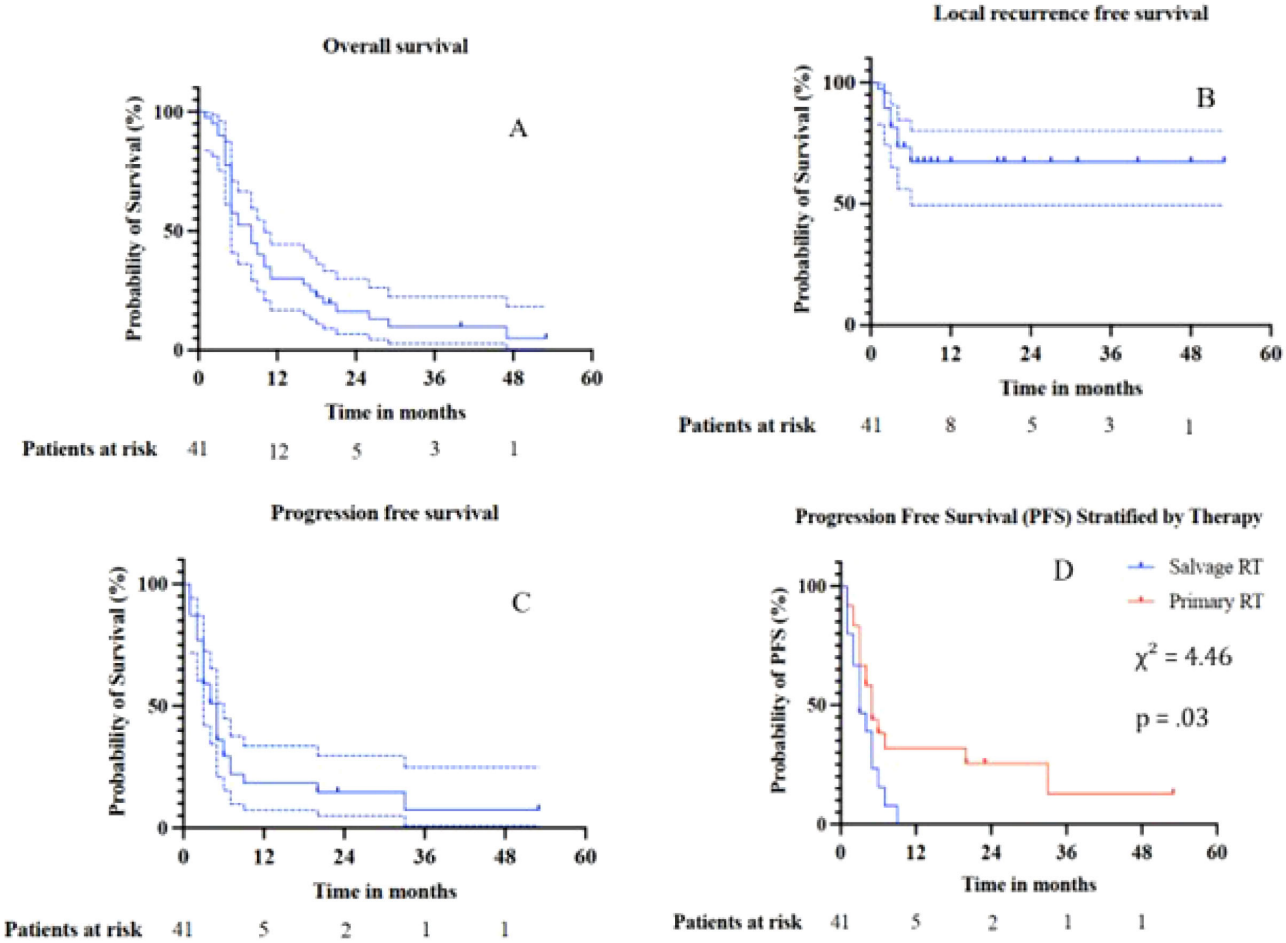
Kaplan-Meier survival curves for overall survival (OS) **(A)**, local recurrence-free survival (LRFS) **(B)**, progression-free survival (PFS) **(C)**, and for lprogression free survival following salvage proton therapy **(D)**. Dashed lines indicate 95% confidence intervals.

41 patients were included in this retrospective analysis. The median age at the start of radiotherapy stood at 69.5 years (range: 49–82, SD = 8.4). The cohort consisted of 24 men (58.5%) and 17 women (41.5%). 29 patients (70.8%) had T3/4 carcinomas. Lymph node involvement was observed in 25 patients (61.0%), and distant metastases were present in one case (2.4%). Thus, the majority of patients (73.2%) were in stage III. 58.5% were classified as unresectable, while 14.6% were seen as borderline resecatble and 26.8% as resectable according to NCCN guidelines (28). All borderline resectable patients had received neoadjuvant Chemotherapy before surgery. The mean CA 19–9 level at baseline (available for 27 patients) was 394.2 U/L (SD = 623.0 U/L). More insights are found in **Table 1**. The one- and two-year OS were 30% (CI 95% 15.7% –33.7%) and 16.4% (CI 95% 2.9% – 26.1%) while the one- and two-year PFS stood at 18.4% (CI 95% 6.6% –33.6%) and 14.7% (CI 95% 5% –29.4%) respectively. All local recurrences occurred within six months, leading to a LFRS of 68.3% (CI 95% 54.6% –85.1%) in our FU-period. The Kaplan-Meier curves are found in **Figure 1**.

**Table 1:** Characteristics of our cohort of 41 patients.

Age at RT, median, range *(SD)*	69, 49 - 82 (± 8.4)
Sex, number (%)	
*Male*	24 (58.5%)
*Female*	17 (41.5%)
T-stadium	
*T1*	2 (4.9%)
*T2*	10 (24.4%)
*T3*	17 (41.5%)
*T4*	12 (29.3%)
N-stadium	
*negative*	16 (39.0%)
*positive*	25 (61%)
M-stadium	
*negative*	40 (97.6%)
*positive*	1 (2.4%)
UICC-stage, number (%)	
I	1 (2.4%)
II	9 (22%)
III	30 (73.2%)
IV	1 (2.4%)
Resectable, number (%)	11 (26.8%)
Borderline Resectable, number (%)	6 (14.6%)
Unresectable, number (%)	24 (58.5%)
CA 19-9, mean (SD) for n = 27	394.2 U/L (± 623.01 U/L)

SD, standard deviation, Union for International Cancer Control, U/L, units per litre, ±, respective means ± SD, TNN-stadiums referring to clinical stadium at the time of RT.

Sixteen patients (39.0%) underwent surgical resection as primary treatment and received salvage RT in case of local recurrence after a median of nine months post-surgery. The remaining 25 patients (61.1%) underwent primary RT or RCx. Radiotherapy was typically delivered in 30 fractions with a median dose per fraction of 1.8 GyE. The median total dose was 54.0 GyE and the median biologically effective dose (BED) was 64.0 GyE. The median BECD (biologically effective chemotherapy dose) was 76.6GyE if a gain in TCP of 0.1 was assumed and 78.1 for an assumed gain of 0.2. A median of four cycles of Gemcitabine was applied per patient. The PTV was accesible for 35 patients, with a median of 228 mL. Most patients received proton therapy (n = 39, 95.1%), and 2 (4.9%) were treated with combined photon and proton therapy. **Table 2** provides additional information regarding the treatment characteristics. 

**Table 2:** Treatment characteristics for the cohort of 41 patients.

Initial treatment, number (%)	
*Salvage RT*	16 (39.0%)
*Primary Surgery only*	9 (22.0%)
*Primary Surgery + neoadjuvant Chemotherapy*	7 (17.1%)
*Primary Radiotherapy*	25 (61.0%)
Time to recurrence after primary surgery, median, range (SD)	9 months, ± 2.6 months, 1–9 months
Radiotherapy characteristics	
*Number of fractions, median, range (SD)*	30 fx, 23–35 fx (± 2 fx)
*Dose per fraction, median, range (SD)*	1.8 GyE, 1.8 GyE – 2.5 GyE (*±* 0.2 GyE)
*Total applied dose, median, range (SD)*	54.0 GyE, 45.0 – 63.0 GyE (*±* 3.4 GyE)
*BED, median, range (SD)*	64.0, 52.6 – 81.3 (*±* 5.4)
*BECD 0.2, median, range (SD)*	78, 65.2 – 95.8 (*±* 6.7 GyE)
*BECD 0.1, median, range (SD)*	76.6, 63.7 – 94.1 (*±* 6 GyE)
*PTV for n =35 cases, median, range (SD)*	228 mL, 91.2 – 4126, 0 mL (*±* 695, 8 mL)
Type of Radiotherapy	
*Proton therapy, number (%)*	39 (95.1%)
*Combined photon and proton therapy*	2 (4.9%)
Chemotherapy	
*Neoadjuvant Chemotherapy, number (%)*	25 (61.0%)
*Concomitant Chemotherapy*	28 (68.3%)
*Concomitant cycles, median, range (SD)*	4, 0 – 6 (± 2, 3fx)
*Adjuvant Chemotherapy*	19 (46.3%)

SD, standard deviation; PTV, planning target volume; mL; milliliters; fx; fraction; BED, biologically effective dose; BECD, biologically effective chemotherapy dose, BECD TCP 0.2/0.1, biologically effective chemotherapy dose with an assumed TCP gain through chemotherapy of 0.2 and 0.1 respectively ±, respective means ± SD.

### Toxicity

Treatment-related toxicity was assessed according to CTCAE v5.0 (and AKIN for renal events) (29, 30) at early (6 weeks post-RT) and late follow-up (≥ 6 months post-RT). During early follow-up, the majority of patients experienced no or only mild toxicity (≤ grade II). One patient (2.4%) died due to acute renal failure (grade V) within one month following RT. However, this patient had a prior history of chronic renal failure which became acute as pre-renal failure weeks after RT. Also, the genitourinary system received <5GyE, so we assume this to be non-RT related. In the late follow-up period, most toxicities had resolved or remained mild. Two patients (4.9%) experienced grade III toxicity, while no grade IV or V events occurred. Overall, PrT was well tolerated, with a low incidence of high-grade acute and late toxicities.

### Data analyses

No significant differences were observed between patients treated with salvage versus primary PrT in age (p = .4), BED (p = .48), CA19-9 (p = .64), or total dose (p = .35). No significant associations were found between treatment group and sex (p = .29), as well as in the application of pre-treatment, (p = .07), concurrent (p = .46), or post-treatment chemotherapy (p = .24), too. In contrast, T-stage differed significantly between groups (χ² (3) = 22.39, p<.001), with lower T stages (T1–2) predominantly observed in the salvage group and higher stages (T3–4) being more frequent in the primary PrT group. This was reflected in the T3/4 classification (χ² (1) = 13.81, p<.001), where T3/4 tumors were notably more common among patients receiving primary radiotherapy. Lymph node involvement also showed significant group differences for both full N staging (χ² (2) = 12.51, p = .002) and dichotomized nodal status (χ² (1) = 4.53, p = .03), indicating more advanced nodal disease in the salvage group.

### Predictors of oncological performance

**Table 3** gives an overview of the statistical results regarding LRFS, PFS and OS for our cohort. Univariate analyses were employed to identify predictors of LRFS. We observed 13 cases of LR in our FU-period, all occurring within six months after RT. Univariate Cox-regression analyses identified pre-treatment CA19-9 (p = .04, HR = 1.00 CI 95% 1.00 –1.00) as theoretical predictor of LR – however the CIs and the HR were 1, indicating this association is clinically insignificant. Apart from that, no other significant associations were identified. Notably, no significant associations between applied dose, including the BECD, previous surgery or Cx with LR were identified. Due to the low event rate, multivariate testing (MVA) was not performed.

**Table 3:** Univariate log-rank and Cox-regression analyses for LRFS, PFS und OS (n = 41 patients).

	LRFS (n = 13 recurrences)				PFS (n = 33 recurrences)				OS (n = 37 deaths)			
	Log rank		Cox-regression		Log rank		Cox-regression		Log rank		Cox-regression	
	χ²	p	p	HR (CI95%)	χ²	p	p	HR (CI95%)	χ²	p	p	HR (CI95%)
Sex	1.06	.30	.6	.32 (.17 - 1.8)	2.06	.15	.19	.62 (.3 –.26)	.51	.47	.50	.79 (.40 - 1, 55)
Age	n/a	n/a	1.1	.26 (.97 – 1.12)	n/a	n/a	.16	1.03 (.99 - 1.08)	n/a	n/a	.17	1.03 (.99 – 1.07)
Salvage RT	.82	.36	1.6	.38 (.55 – 4.86)	**4.46**	**.03**	.06	2.00 (.98 – 4.07)	.08	.77	.79	1.10 (.56 – 2.16)
UICC-stage	n/a	n/a	.27	2, 00 (.59 – 6.78)	n/a	n/a	.58	1, 22 (.6 – 2.46)	n/a	n/a	.77	1.09 (.61 – 1.94)
Nodal stage	0.64	0.47	.9	.93 (.3 – 2.87)	1.05	0.3	.34	1.44 (.67 – 3.1)	.74	.39	.41	.76 (.39 – 1.48)
CA 19-9	n/a	n/a	.**04**	**1.00** **(1.00 –1.00)**	n/a	n/a	**.01**	**1.0 (1.0 – 1.0)**	n/a	n/a	.06	1.00 (1.00 –1.00)
BED	- n/a	n/a	.13	1, 10 (.97 – 1.24)	n/a	n/a	.54	1.02 (.96 – 1.09)	n/a	n/a	.37	1.03 (0.97 – 1.10)
BED75	.39	.54	.39	.74 (.37 – 1.5)	.01	.93	.93	.97 (.46 – 2.1)	.81	.39	.39	.72 (.46 – 1.48)
BED80	1.46	.23	.89	1.05 (.51 – 2.16)	.04	.95	.95	.98(.46 – 2.1)	.02	.88	.24	1.7 (.69 – 4.2)
BECD TCP 0.2	n/a	n/a	.2	1.05 (.97 – 1.14	n/a	n/a	.72	1.01 (.95 – 1.07)	n/a	n/a	.0.6	1.05 (.96 – 1.07)
BECD TCP 0.1	n/a	n/a	.18	1.06 (.98 – 1.15)	n/a	n/a	.63	1.02 (.96 – 1.08)	n/a	n/a	.0.5	1.02 (.96 – 1.08)
PTV	n/a	n/a	0.3	.9 (.9 – 1.1)	n/a	n/a	.30	1.00 (1.00 – 1.00)	n/a	n/a	.19	1.00 (1.00 –1.00)
Neoadj. Cx	.90	.34	.36	.60 (.2 - 1.8=	3.55	.06	.19	.64 (.33 - 1, 25)	1.90	.17	.19	.64 (.33 -1.25
Conc. Cx	.06	.80	.81	.86 (.27 – 2.80)	.01	.91	.50	.79 (.39 - 1, 59)	.49	.48	.5	.79 (.39 – 1.59)-
Adj. Cx	.00	.96	.96	.97 (.33 – 2.91)	2.35	.13	.08	.53 (.26 - 1, 08)	3.41	.06	.08	.53 (.26 – 1.08

χ2 (1), chi square value with one degree of freedom; p, p-value; HR, hazard ratio, CI confidence interval, UICC-stage, Union for International Cancer Control staging, nodal stage, lymph node involvement, CA 19-9, carbohydrate antigen 19-9, BED=biologically effective dose, BECD=biologically effective chemotherapy dose, BED75/BED80, biologically effective dose of at least 75 or 80GyE, BECD TCP 0.2/0.1, biologically effective chemotherapy dose with an assumed TCP gain through chemotherapy of 0.2 and 0.1 respectively, PTV, planning target volume; Neoadj. Cx, neoadjuvant chemotherapy; Conc. Cx, concurrent chemotherapy; Adj. Cx, adjuvant chemotherapy.

Significant results highlighted as bold values.

Meanwhile, 33 overall progressions were observed with 31 occurring during the first year of FU. In 20 cases, these progressions were solely due to distant metastases. Here, salvage RT was revealed as predictor of worse PFS (χ² = 4.46p = .03) per Log-rank test, although falling short of significance per Cox-regression (p = 0.06). Again, CA-19–9 was (p = .01, HR = 1.00 CI 95% 1.00 –1.00) significant, but the same reasoning applies to its clinical significance. On MVA, neoadjuvant chemotherapy (HR = .65, p = .28 95% CI:.30–1.42), adjuvant chemotherapy (HR = 0.70, p = .35, 95% CI:.34–1.48) and salvage RT (HR = 1.70, p = .17, 95% CI: 0.80–3.61) failed to show a significant association with PFS.

Lastly, we seeked to identify predictors of OS. 37 patients died in the FU-period with 29 cases dying within the first year after RT. In this instance, no predictors reached statistical significance so that we refrained from MVA. Only trend wise was adjuvant chemotherapy associated with improved OS, as indicated by both log-rank testing (χ² = 3.41, p = .06) and Cox regression (HR = 0.53, 95% CI: 0.26–1.08, p = .08). Notably, mFOLFIRINOX was only administered in 4/19 cases with adjuvant Cx while most patients received Gemcitabine (11/19).

## Discussion

Conventional photon therapy to the Pancreas is limited by proximity of organs at risks (OAR) (12) leading to interest in advanced modalities such as IMRT, SBRT, and particle therapy (14, 31, 32). Particle therapy, encompassing both proton therapy and carbon ion therapy, offers distinct physical and biological advantages over conventional photon radiation (31). The principle underlying these advantages is in the unique energy deposition pattern of charged particles, characterized by the Bragg peak phenomenon. Unlike photons, protons and heavier ions deposit minimal energy at the entrance, reach maximum energy deposition at a specific depth (the Bragg peak) and then rapidly fall to zero beyond the target (13).

The elevated relative biological effectiveness (RBE) of proton therapy compared to photon therapy adds another dimension to its therapeutic potential (24). Recent consensus statements from the Particle Therapy Cooperative Group have emphasized the potential benefits of proton beam therapy for pancreatic tumors, particularly in reducing toxicities compared to photon therapy while maintaining or improving therapeutic efficacy (33). A recent single-center retrospective study by Seto et al. reported encouraging outcomes for proton beam therapy in unresectable locally advanced pancreatic cancer (34). In their cohort of 54 patients treated with a median dose of 67.5 GyE, they achieved a median overall survival of 18.2 months, with 1-year and 2-year overall survival rates of 77.8% and 35.2%, respectively. Notably, their study demonstrated excellent local control, with 1-year and 2-year local progression-free survival rates of 89.7% and 74.5%, respectively. Similar results were shown by Kim et al. (35), however doses to the GI-system (e.g. stomach or duodenum) have traditionally been dose-limiting. While PrT cannot fully resolve this, existing data suggests that it can reduce the dose to healthy tissues in comparison with photon RT, especially for smaller tumors (36, 37) thus allowing necessary dose escalation.

Compared to these results, our cohort’s outcomes appear modest, though direct comparison is challenging due to differences in patient selection, treatment protocols, and follow-up periods. The Seto study’s superior outcomes may be attributed to their use of higher radiation doses (median 67.5 GyE vs. our median 54.0 GyE) and more consistent use of concurrent chemotherapy (85.2% vs. our 68.3%). The dose-response relationship observed in the Seto study is particularly noteworthy, with patients receiving ≥67.5 GyE demonstrating significantly better local progression-free survival compared to those receiving lower doses (96.7% vs. 77.9% at 1 year, p = 0.015) (34). This finding supports the hypothesis that pancreatic cancer benefits from dose escalation, a concept that has been consistently demonstrated in photon-based studies as well (21, 34). Similar results were achieved in Terashima’s work, who reached a one-year OS and LRPFS of 81.7% and 64.3% respectively for proton based RCx with Gemcitabine for 50 patients. Here, most patients received dose-escalated therapy up to 67.5 GyE – 70.2 GyE (17). The moderate prescribed dose in our cohort (median 54 GyE, BED 64 GyE) reflected the absence of daily CT-based image guidance in our facility. The need to rely on X-ray imaging as sole method for IGRT worsens the ability to clearly differentiate between OARs and target volumes in the upper GI-tract. For the treatment of PC, this is of special concern since radiosensitove structures like the Duodenum, Jejunum and Ileum are often in close proximity or even adjacent to the target volume. The lack of daily CT-scan therefore led us to rely on doses that would more easily be tolerated by surrounding OARs and take away the ability to employ concepts like internal protections to those OARs, which are increasingly employed in SBRT (38). Our findings, however, indicate that this strategy led to limited local control. With modern IGRT, escalation toward ≥67.5 GyE should be mandated and such modern IGRT appears as fundamental to the treatment of PC.

A significant finding of our study is the favorable toxicity profile observed with proton therapy. The low incidence of high-grade acute and late toxicities aligns with the theoretical advantages of proton therapy and supports its clinical application in pancreatic cancer. Our observation of only one case of severe early toxicity (2.4%), likely unrelated to the RT, and two cases of late grade III toxicity (4.9%) compares favorably with historical data from conventional radiation therapy series. The Seto study reported a similar toxicity profile, with acute hematologic toxicities being the most common adverse events (34). Importantly, they observed no late adverse events of grade 3 or higher, which is particularly significant given the proximity of critical organs to the pancreas. This finding is consistent with dosimetric studies demonstrating significant reductions in dose to OARs with proton therapy compared to photon-based techniques. The favorable toxicity profile observed with proton therapy has important implications for treatment intensification strategies. The ability to deliver higher doses while maintaining acceptable toxicity levels opens the possibility for dose escalation protocols that may improve local control and potentially overall survival. This is particularly relevant given the dose-response relationships observed in multiple studies. In Terashima’s work however, dose escalation could not be performed in 12% of patients due to hemorrhages and ulcers and 10% had late ≥ °III toxicity, possibly due to increased dose at the adjacent OARs (17).Therefore, the right balance is yet to be found and IGRT has a pivotal role in allowing this dose escalation. Our work however seems to indicate the opposite: Lesser RT-doses, which likely led to the low incidence of severe AEs in this study, may come at the cost of lesser tumor control. Given the overall scarcity of data, joint efforts seem to be key to further elucidate whether RT-dose is the main predictor of higher AE-rates or if other predictors exist that could aid to guide patients to dose escalated or to more conservative RT-approaches.

In our collective, salvage RT was a predictor of lesser PFS which could be expected as tumor control and survival rates generally drop in the case of recurrent disease. Adding to this, although some promising data for the use of particle therapy exists, surgery is the more established and likely more successful alternative (39, 40). Furthermore, the same reasoning as discussed above applies for this subgroup. Both Kawashiro et al. and Liermann et al. have demonstrated better results with dose escalation via C12-therapy (39, 40) with one year LC-rates of 87.5% and 69%. Similar results were presented in a recent SBRT-study (41).

As the lack of significant association between applied dose and local recurrence in our study contrasts with findings from other series, an aim of our study was to analyze, whether concomitant Cx can compensate for lesser RT-doses, as reflected by our BECD calculation, thus allowing lesser dose at OARs while maintaining oncological results. Interestingly though, we could not identify such an influence for both TCP-rates assumed. This leaves the question unanswered, which doses of Radiotherapy and Chemotherapy are necessary to lead to a better prognosis. Clearly however, Gemcitabine alone seems insufficient to compensate for lower RT-doses. Therefore, should Gemcitabine be used, we do not encourage dose de-escalation. For other regimens, similar approaches could aid to find out if higher RT-doses remain to be mandated or not or if an even greater tumor control could be achieved through escalation of both chemo- and radiotherapy.

Another result in line with previous findings is that the main driver of progression are distant metastases (17, 34, 39–41). While the wish for improved local control is undeniable, gaining systemic tumor control seems imperative. The trend toward improved overall survival with adjuvant chemotherapy, while not reaching statistical significance in our study, aligns with established treatment paradigms and the growing body of evidence supporting intensified systemic therapy to achieve that. In comparison, Gemcitabine may not be sufficient to control microscopic metastatic disease. Our observation that only 4 of 19 patients receiving adjuvant chemotherapy received mFOLFIRINOX, with the majority receiving gemcitabine-based regimens, may have limited the potential survival benefit as the recent success of mFOLFIRINOX in the adjuvant setting has established this chemotherapy as a standard component of pancreatic cancer treatment (12). Right now, the ideal combination of radio- and chemotherapy is unclear. However, chemotherapy regimens have evolved greatly over the years and both induction chemotherapy before radio chemotherapy and novel combinations of radio chemotherapy should be considered. On one hand, trials like SCALOP demonstrate promising results with induction Chemotherapy before radiotherapy for unresectable PC (42) and the use of neoadjuvant FOLFIRINOX has proven its potential in combination with proton radio chemotherapy with capecitabine in the case of borderline resectable PC (43). At the same time, the administration of radio- and chemotherapy remains confusing: Our trial, as did Seto’s (34), reports too many combinations of different neoadjuvant chemotherapy regimens (and in the latter case even differing concomitant ones) to clearly determine who could benefit not only from escalation of the radiotherapy dose, but also of an escalation of chemotherapy dose or an intensification of chemotherapy regimens. While early data suggests that dose escalated radiotherapy in conjunction with nab-Paclitaxel is feasible (44), efficacy is still unclear and FOLFIRINOX is only routinely used prior to radiotherapy.

All this should lead us to two conclusions: Firstly, the therapy regimens used for unresectable or borderline resectable PC should be harmonized. Neoadjuvant treatments before radiotherapy should be encouraged unless excess toxicity is to be expected. Likewise, radiotherapy doses should be escalated, be it with protons or SBRT. We suggest understanding both as means to reduce OAR-doses while escalating tumor dose and the decision between both should be based on individual tumor sizes, anatomy and the anticipated target volume. Secondly, multi-institutional efforts should be made to gain pooled data for the overall rare cases of PC to determine the most effective and safest combination of neoadjuvant, concomitant and possibly consolidating radio chemotherapy.

Several limitations of our study must be acknowledged when interpreting these results. The retrospective nature of the analysis introduces selection bias and limits the ability to control for confounding variables. The small sample size of 41 patients, while reasonable for a single-center proton therapy study, limits the statistical power for detecting significant associations and may not be representative of the broader pancreatic cancer population. The heterogenic treatment indications (primary vs. salvage RT), treatment protocols including variations in radiation dose, surgery, fractionation schedules, and chemotherapy regimens, reflect the evolution of treatment approaches over the 12-year study period but complicate the outcome interpretation. The lack of a control group receiving conventional photon therapy prevents direct comparison of treatment modalities and limits conclusions about the relative efficacy of proton therapy.

Despite that, our study provides valuable real-world evidence for the safety and feasibility of proton therapy in pancreatic cancer. The favorable toxicity profile supports the continued investigation of proton therapy, particularly in the context of dose escalation protocols. The dose-response relationships observed in contemporary literature suggests that future protocols should incorporate higher radiation doses, potentially in the range of 67.5–70 GyE, to optimize local control. Also, efforts should be made to harmonize the integration of Cx in the RT-protocols. The integration of advanced imaging techniques, including MRI-guided adaptive radiation therapy, may further enhance the precision of proton therapy delivery and allow for safe dose escalation. The development of standardized protocols for biologically effective dose calculations, as attempted in our study with the BECD methodology, represents a step toward optimizing combined modality treatments. However, further validation of these models, *in vitro* and *in vivo*, will be necessary before widespread adoption.

## Conclusion

This retrospective analysis of 41 patients with pancreatic cancer treated with proton therapy at the Marburg Ion Beam Treatment Center provides valuable real-world evidence for the safety and feasibility of this advanced radiation technique in a challenging clinical setting. Our findings contribute to the growing body of literature supporting the role of proton therapy in the multidisciplinary management of pancreatic cancer. Strikingly, they demonstrate the need for dose escalated RT and modern IGRT facilitating the application of higher RT-doses. Novel radio chemotherapy concepts should further be tested as alternatives to gemcitabine, given the great advances of systemic therapy in recent years.

## Data Availability

The data analyzed in this study is subject to the following licenses/restrictions: The dataset consits of patients treated at our institution. A blinded version of the data may be provided on request. Requests to access these datasets should be directed to Philipp Lishewski, lishewsp@staff.uni-marburg.de.
