# Supplementary material for: Proton therapy for pancreatic cancer: real-world single-center experience of efficacy, toxicity, and predictors of outcome
Source: Front Oncol. 2026 Feb 26;16:1710943. doi: 10.3389/fonc.2026.1710943 (PMC12979228; doi:10.3389/fonc.2026.1710943)
Supplement: Supplementary file 1 [file Table1.docx]

Supp. table 1: Primary Treatment characteristics for the cohort of 41 patients

Primary treatment	
Surgery, total	16 (39.0%)
Surgery only	9 (22.0%)
Surgery + neoadjuvant Chemotherapy	7 (17.1%)
Resection with positive margins	3 (7.3%)
Radiotherapy, total	25 (61.0%)
Radiochemotherapy	16 (64.0%)

SD = standard deviation, ± = respective means ± SD.
